# Pharmacokinetics and Tissue Distribution of Anwuligan in Rats after Intravenous and Intragastric Administration by Liquid Chromatography-Mass Spectrometry

**DOI:** 10.3390/molecules25010039

**Published:** 2019-12-20

**Authors:** Yang Song, Yuan Zhang, Xiao-Yi Duan, Dong-Wei Cui, Xin Qiu, Yu Bian, Ke-Fei Wang, Xue-Song Feng

**Affiliations:** 1 School of Pharmacy, China Medical University, Shenyang 110122, China; songyanglhyb1998@163.com (Y.S.); dxychn@126.com (X.-Y.D.); cdwhhazj@163.com (D.-W.C.); 13314227291@163.com (X.Q.); bianyu2468346755@126.com (Y.B.); wl5712367805@sina.com (K.-F.W.); 2 Department of Pharmacy, National Cancer Center/National Clinical Research Center for Cancer, Chinese Academy of Medical Sciences and Peking Union Medical College, Beijing 100730, China; 13840149878@163.com

**Keywords:** anwuligan, LC-MS/MS, pharmacokinetics, tissue distribution, rat

## Abstract

Anwuligan, a natural 2,3-dibenzylbutane lignan from the nutmeg mace of *Myristica fragans*, has been proved to possess a broad range of pharmacological effects. A rapid, simple, and sensitive liquid chromatography tandem mass spectrometry (LC-MS/MS) method has been established and successfully applied to the study of pharmacokinetics and tissue distribution of anwuligan after intravenous or intragastric administration. Sample preparation was carried out through a liquid-liquid extraction method with ethyl acetate as the extraction reagent. Arctigenin was used as the internal standard (IS). A gradient program was employed with a mobile phase consisting of 0.1% formic acid aqueous solution and acetonitrile. The mass spectrometer was operated in a positive ionization mode with multiple reaction monitoring. The transitions for quantification were *m*/*z* 329.0→205.0 for anwuligan and *m*/*z* 373.0→137.0 for IS, respectively. Calibration curves were linear over the ranges of 0.5–2000 ng/mL for both plasma samples and tissue samples (r > 0.996). The absolute bioavailability is 16.2%, which represented the existing of the obvious first-pass effect. An enterohepatic circulation was found after the intragastric administration. Anwuligan could be distributed rapidly and widely in different tissues and maintained a high concentration in the liver. The developed and validated LC-MS/MS method and the pharmacokinetic study of anwuligan would provide reference for the future investigation of the preclinical safety of anwuligan as a candidate drug.

## 1. Introduction

Anwuligan is a natural 2,3-dibenzylbutane lignan from the nutmeg mace of *Myristica fragans* [1,2]. *Myristica fragans* (*M. fragrans*) is a tropical evergreen tree native to Indonesia and cultivated in India, Iran, the West Indies and South America. The mace found in *M. fragrans* has been reported to have anti-fungal and anti-bacterial activities [3]. Anwuligan has been proved to possess a broad range of pharmacological effects (see Table S1), including anti-bacterial, anti-inflammatory, and anti-cancer activities. Recently, it has been proved to have anti-diabetic, hepatoprotective, and neuroprotective effects. In terms of antibacterial activity, anwuligan could inhibit the growth of various bacteria, including *Bacillus cereus* [4], *Streptoccus* bacteria [5], *Lactobacillus* bacteria [5]; it could also effectively inhibit oral colonizing bacteria and has the potential to remove these bacteria and reduce the plaque formation [6]. In terms of anti-cancer activity, anwuligan could induce apoptosis of human promyeloytic leukemia cells (HL-60) by activating caspase-3 [7], it also exhibits the anti-carcinogenic activity by strongly inhibiting the carcinogenic oral bacteria *S. mutans* [5] and ameliorating the side effects of cancer chemotherapy by regulating the activity of multidrug-resistant protein [8,9,10]. In terms of hepato-protection activity, anwuligan has been proved to be a drug-metabolizing enzyme (DME) modifie, possessing the ability to inhibit aminopyrin-*N*-demethylase [11] and ameliorate the cytotoxicity induced by tert-butyl hydroperoxides (*t*-BHP) in human hepatoma cells (HepG2) [1], anwuligan also has a protective effect on cisplatin-induced hepatotoxicity, which is related to the mitogen activated protein kinase (MAPK) signaling pathway [12]. In terms of anti-diabetic function, anwuligan has an insulin secretagogue action and maintains blood glucose level by inhibiting the intestinal ALPHA-glucoamylase [13], it is also a natural peroxisome proliferator-activated receptor (PPAR)α/γ dual agonist contributed to the complementary and synergistic increases in lipid metabolism and insulin sensitivity [14]. In addition, anwuligan acts on dermalogical protection and has the potential for the treatment of abnormal pigmentation, aging, and related skin diseases [15,16]. In terms of anti-oxidative and anti-inflammatory activities, anwuligan protects mammalian splenocytes from radiation-induced intracellular reactive oxygen species (ROS) production [17]; it also reduces the expression of pro-inflammatory cytokines, TNF (tumor necrosis factor)-α, IL-6, IL-1b, and C-reactive protein [18] and inhibits histamine degranulation [19]. Anwuligan was reported to inhibit the neurotoxicity of glutamate on HT22 cells in the hippocampus of murine and the production of corticotrophin, and have an effect on inflammatory memory loss in chronic LPS rats showing the potential to treat neurodegenerative diseases [20,21]. Toxicity studies have shown that anwuligan has no cytotoxic effect on lymphocytes in vitro [17].

Based on the above results of pharmacological and toxicological studies, it could be inferred that anwuligan is a candidate drug for the treatment of many diseases, which represents a new potential pathway for natural intervention and shows advantages over synthetic chemistry. As far as we know, the study of anwuligan analysis are mainly on HPLC and UV method [22,23], and both are in vitro content determination studies. At present, the pharmacokinetics and tissue distribution of anwuligan have not been reported. It is generally acknowledged that the main purpose of LC-MS bioanalysis is to provide quantitative methods for the candidate drugs, so as to come to accurate and reliable conclusions of pharmacokinetic and tissue distribution studies. Therefore, this study is to establish an accurate and reliable LC-MS/MS method for the determination of anwuligan in rat plasma and tissues, and to be successfully applied in the pharmacokinetic and tissue distribution study, which would provide data support and reference for the future evaluation of preclinical safety of anwuligan as a candidate drug.

## 2. Results

### 2.1. Method Validation

#### 2.1.1. Specificity

**Figure 1:**
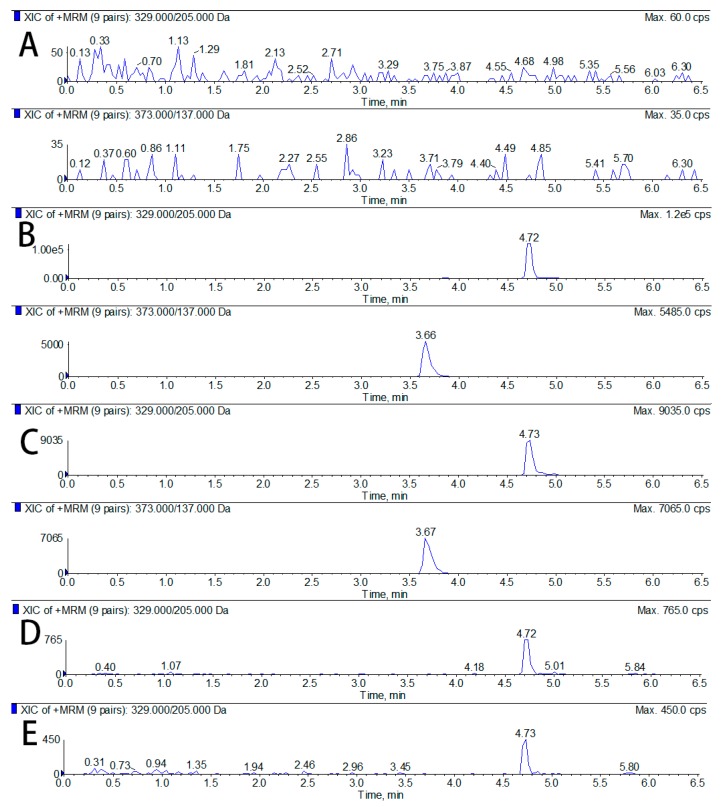
Representative MRM chromatograms of anwuligan: (**A**) blank plasma; (**B**) blank plasma spiked with anwuligan and IS; (**C**) plasma sample collected 1h after oral administration of anwuligan (60 mg/kg); (**D**) blank plasma spiked with anwuligan (LLOQ); and (**E**) blank liver tissue homogenates with anwuligan (LLOQ).

As shown in Figure 1, the retention time for anwuligan and IS were 4.72 and 3.66 min, respectively. No endogenous interference was observed at the retention time of anwuligan and IS.

#### 2.1.2. Calibration Curve and LLOQ

The calibration curves were calculated by a linear regression model for plasma and tissue samples. As shown in Table 1, the linear concentration range was from 0.5 ng/mL to 2000 ng/mL for both plasma and tissue samples, with the coefficient values all more than 0.99.

#### 2.1.3. Precision and Accuracy

Table 2 shows the results of intra-day and inter-day precision and accuracy of anwuligan in rat plasma and tissues. The precision values expressed as RSD were all within 11.3% and the accuracy values expressed as RE ranged from −12.6% to 10.6%, indicating that the method was accurate and reproducible to the quantitative determination of anwuligan in rat plasma and tissues.

#### 2.1.4. Extraction Recovery and Matrix Effect

As presented in Table 3, the extraction recoveries for anwuligan were within 84.0%–114.5%, and the matrix effects for anwuligan were within 84.5% to 96.8%. The results indicated that the extraction efficiency was high and there was little matrix effect for the analyte.

#### 2.1.5. Stability

Table 4 shows the results of stability of anwuligan under the four given storage conditions, which indicated there was no stability issue occurred.

#### 2.1.6. Dilution Intergrity

Dilution integrity was assessed by six replicate samples, with the plasma concentration of 20.0 μg/mL and tissue homogenate concentration of 12.5 μg/mL (50.0 μg/g tissue) for anwuligan, were diluted 20-fold with blank rat plasma and blank tissue homogenate respectively, which was within the range of standard curve. The precision (CV) was less than 15% and the accuracy was within 85%–115% for the analyte.

### 2.2. Dose Selection

The intravenous administration dose was generally determined based on 1/20–1/50 of the LD50 value or 1/2–1/3 of the maximum tolerated dose of the reference MTD [24,25,26]. The LD50 obtained by our previous pharmacological experiment was 573 mg/kg and therefore the selected i.v. dose was 5 mg/kg. Meanwhile, it is reported that the structural analog of anwuligan, was given to rat i.v. at a dose of 20 mg/kg for pharmacokinetic study [27,28], was given to rat i.g. at a dose of 40 mg/kg for pharmacokinetic study [28]. The intravenous dosage of another piece of literature is 20 mg/kg for pharmacological activity study [29]. Above all, the dosage of drug intravenous and intragastric administration in our pharmacokinetics experiment is determined to be 15 mg/kg and 60 mg/kg, respectively. In addition, the bioavailability of different routes of administration is different. Generally, the bioavailability of intravenous administration is 100%, oral administration is about 25%~30%, and the ratio is 1/4. The calculated bioavailability was 16%, which was close to 25%, so the ratio of the two routes was more reasonable.

### 2.3. Pharmacokinetic Study

**Figure 2:**
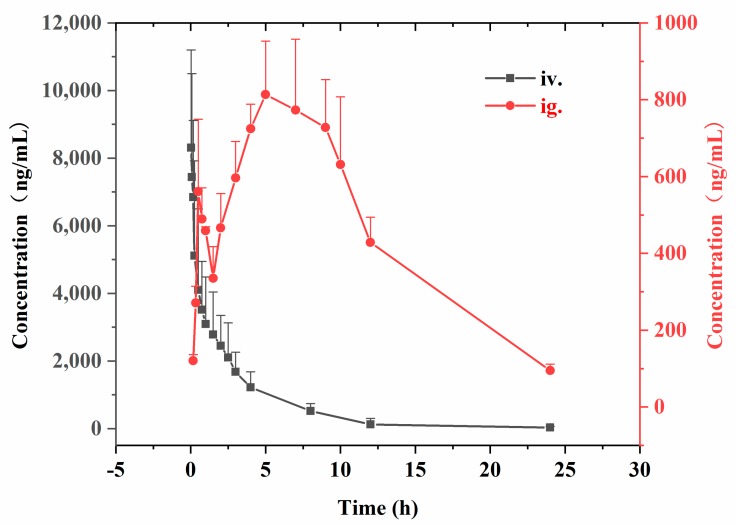
The plasma concentration-time course in rats by given i.v. dose of 15 mg/kg of anwuligan (*n* = 6) and given i.g. dose of 60 mg/kg of anwuligan (*n* = 6).

The validated LC-MS/MS method has been successfully applied to the pharmacokinetics and tissue distribution study of anwuligan after the intravenous or intragastric administration. The mean plasma concentration–time profile of anwuligan after administration was shown in Figure 2. The pharmacokinetic parameters based on the non-compartmental method were summarized in Table 5.

#### 2.3.1. Intravenous Administration PKs

Upon intravenous administration of 15 mg/kg anwuligan, the C_max_ was 8310 ± 910 ng/mL, t_1/2_ was 3.20 ± 1.09 h, indicating that anwuligan was rapidly eliminated in plasma. The pharmacokinetic results have demonstrated the area under the curve up to the last sampling time (AUC_0–t_) and the infinite time (AUC_0−∞_) of 16,700 ± 7700 and 16,800 ± 8500 ng/mL∙h, respectively. The total mean residence time up to the last sampling time (MRT_0–t_) was found to be 3.71 ± 0.96 h. Anwuligan had an apparent V_d_ of 4.1 ± 1.1 L/kg which showed that the drug had no accumulation in tissues.

#### 2.3.2. Intragastric Administration PKs

Upon intragastric administration of 60 mg/kg anwuligan, the C_max_ was 810 ± 190 ng/mL. The drug was rapidly absorbed and the t_1/2_ was 5.17 ± 1.82 h, indicating that the drug was relatively slowly eliminated. The pharmacokinetic results have demonstrated the area under the curve up to time (AUC_0−t_) and the infinite time (AUC_0−∞_) of 10,700 ± 2800 and 11,400 ± 3600 ng/mL∙h, respectively. Anwuligan had an apparent V_d_ of 39.3 ± 12.6 L/kg and CL of 5.26 ± 1.39 L/h/kg. The total mean residence time up to time (MRT_0–t_) was found to be 10.02 ± 2.04 h. The absolute bioavailability is 16.2%, which represented the existing of the obvious first-pass effect. As shown in the Figure 2, the drug concentration increased again after 2 h in the oral administration mode, presumably an enterohepatic circulation.

### 2.4. Tissue Distribution

**Figure 3:**
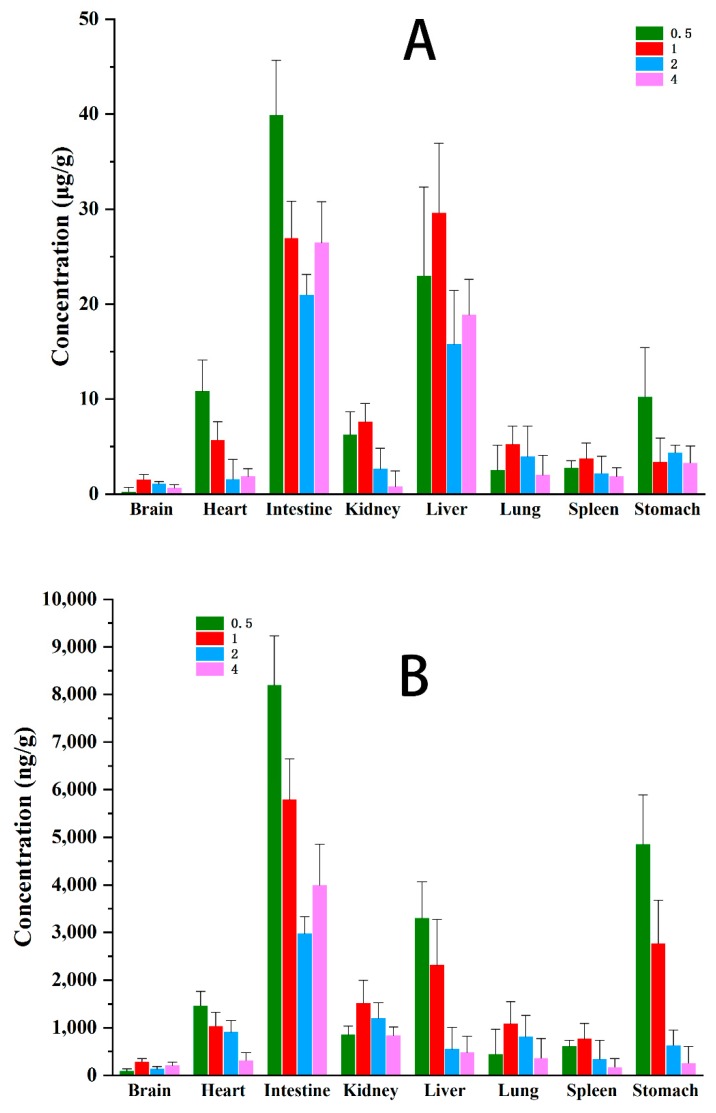
Mean concentration of anwuligan in various tissues at 0.5, 1, 2 and 4 h (**A**) given i.v. dose of 15 mg/kg of anwuligan (*n* = 4) and (**B**) given i.g. dose of 60 mg/kg of anwuligan (*n* = 4).

The change trend of anwuligan concentration in different tissues at different time points at 0.5, 1, 2, and 4 h after administration was observed. As shown in Figure 3, anwuligan was distributed widely and rapidly in different tissues.

#### 2.4.1. Intravenous Administration Tissue Distribution

In brain, lung, and spleen, the maximum concentration appeared at 1 h, while in kidney, heart, liver, intestine, and stomach tissues, the maximum concentration appeared at 0.5 h. Among all the tissues, the liver and intestine showed the highest average concentration, which was just a proof for the enterohepatic circulation thought in pharmacokinetic study.

#### 2.4.2. Intragastric Administration Tissue Distribution

The maximum concentration of stomach, kidney and intestine tissue appeared at 1 h, the maximum concentration of heart and lung tissue appeared at 2 h, while the maximum concentration of brain and liver tissue appeared at 4 h. Among all the tissues, the average concentration of liver, stomach and intestine was the highest, which proved that there was an enterohepatic circulation. The high concentration of drugs in the stomach and intestine was due to the oral administration mode. Therefore, anwuligan maintained a high concentration in the liver, suggesting anwuligan might undergo a relative slow elimination and long accumulation in the liver. It was reported anwuligan could inhibit cyp450 activity [10] leading to weaken the activity of drug enzymes and slow down the metabolism of their own or other drugs, which could well explain the slow elimination and long accumulation of anwuligan in the liver. Moreover it was reported that the existing of methylenedioxyphenyl moiety in anwuligan indicated it might have the ability in inhibiting aminopyrine-N-demethylase [11], suggesting a plausible hepatoprotective mechanism. Therefore, liver was very likely to be the target organ of curative effect of anwuligan, which requires systematic research in the future. In contrast, anwuligan was less likely to cause drug accumulation and immune side effects in heart, kidney, brain, lung, and spleen due to the rapid elimination and less drug accumulation. At the same time, anwuligan could be detected in the brain, which indicated that it was able to pass through the blood-brain barrier.

## 3. Materials and Methods

### 3.1. Reagents and Materials

**Figure 4:**
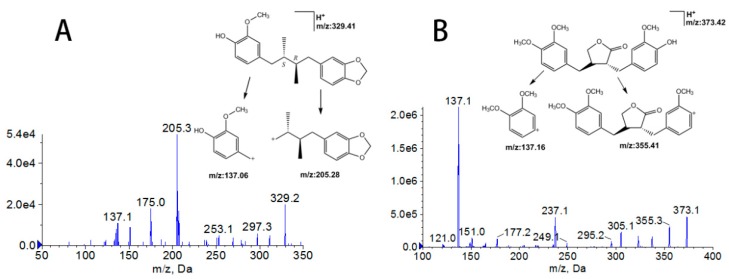
Product ion spectra of [M + H]^+^ for anwuligan (**A**) and arctigenin (IS, **B**).

Anwuligan (purity over 99%) and arctigenin (IS, purity over 99%) were purchased from Chengdu Biopurity Phytochemicals Ltd. (Chengdu, Sichuan. China). Acetonitrile and methanol were MS-grade reagents, purchased from Merck KGaA Company (Darmstadt, Germany) and formic acid was HPLC-grade, purchased from Dikma Company (Lake Forest, CA, USA). Deionized water was purified via a Millipore Milli-Q system (Millipore, Bedford, MA, USA). The other chemical reagents were of analytical grade. The chemical structures of anwuligan and IS are shown in Figure 4.

### 3.2. Animals

Adult male Sprague–Dawley rats (SD, 200 ± 20 g) were obtained from Experimental Animal Research Center, China Medical University (Shenyang, China) and housed in a temperature and humidity controlled room at 24 ± 2 °C and 50 ± 10%. Water and food were available ad libitum. The rats were fed for two weeks and fasted for 12 h before treatment. The whole experimental protocol was approved by the Institutional Animal Care and Use Committee at China Medical University (CMU2019194).

### 3.3. LC-MS/MS Conditions

The biological samples were analyzed using an Agilent series 1290 UHPLC system (Agilent Technologies, Santa Clara, CA, USA) equipped with an AB 3500 triple quadrupole mass spectrometer (AB Sciex, Ontario, ON, Canada) and an electrospray ionization (ESI) source. The separation was carried out on an ACQUITY UPLC BEH C_18_ Column (100 mm × 2.1 mm, 1.7 μm) from Agilent Technologies (Santa Clara, CA, USA) at 30 °C. A gradient program was employed with a mobile phase consisting of 0.1% formic acid aqueous solution (A) and acetonitrile (B) at the flow rate of 0.3 mL/min: 0.0–1.0 min (80%A), 1.0–1.5 min (80%–5%A), 1.5–5.0 min (5%–1%A), 5.0–6.0 min (1%A), 6.0–6.5 min (80%A). The total run time was 6.5 min. The injection volume was 10 μL.

The mass spectrometer was operated in a positive ionization mode, using multiple reaction monitoring (MRM) with following operation parameters: Ionspray Voltage, 5500 V; the turbo spray temperature, 500 °C; ion source gas 1, 19; ion source gas 2, 19; curtain gas, 10. Collision cell exit potential (CXP) and entrance potential (EP) was set at 7.0 V and 10.0 V, respectively. Based on the mass scan and product ion scan (Figure 4), the transitions for quantification were *m*/*z* 329.0→205.0 for anwuligan and *m*/*z* 373.0→137.0 for IS, respectively. Moreover, the qualifier ions for anwuligan and IS were set at *m*/*z* 137.0 and *m*/*z* 355.0, respectively. The declustering potential (DP) for anwuligan and IS were 80 V and 80 V; the collision energy (CE) were 18 eV and 15 eV, respectively. The 1.6.3 version Analyst software package (AB Sciex, Ontario, ON, Canada) was used for data acquisition and instrument control.

### 3.4. Preparation of Calibration Standards and Quality Control (QC) Samples

The stock solution of anwuligan (100 μg/mL) was prepared in acetonitrile, and serially diluted to the working solutions with acetonitrile. The IS working solution with a concentration of 250 ng/mL was also prepared in acetonitrile. The plasma calibration standards were prepared by spiking 50 μL of the working solutions of anwuligan and 50 μL of IS (250 ng/mL) into 100 μL rat blank plasma (or blank tissue homogenate). The final concentrations of anwuligan in rat plasma and tissue homogenates were both ranged from 0.5 to 2000 ng/mL (0.5, 1, 4, 10, 40, 160, 400, 800, and 2000 ng/mL). Quality control (QC) samples at 0.5, 5, 100, and 1600 ng/mL were also prepared in the same manner.

### 3.5. Sample Preparation

#### 3.5.1. Plasma Samples

In this study, a liquid-liquid extraction method was applied to prepare the PKs and tissue distribution samples. An aliquot 100 μL of plasma, 50 μL of IS solution (250 ng/mL) and 1 mL of extraction agent ethyl acetate was added into a 1.5 mL Eppendorf tube. The mixture was vortexed for 1 min. After the centrifugation at 12,000× *g* for 7 min at 4 °C, a 900 μL aliquot of the upper organic layer was carefully transferred and evaporated to dryness at 40 °C under nitrogen. The residuals were reconstituted in 100 μL of initial mobile phase (acetonitrile-0.1% fomic acid water, 20:80, *v*/*v*) by vortex mixing for 30 s. A volume of 10 μL of the supernatant was injected into the LC-MS/MS system for analysis after centrifuging at 12,000× *g* for 10 min.

#### 3.5.2. Tissue Samples

The rats were sacrificed on the ice. The rat tissues were quickly collected, including the heart, the liver, the spleen, the lung, the kidney, the brain, the stomach, and the intestine, and washed with ice-cold 0.9% NaCl to remove the surface blood. After suction with filter paper, a certain amount of tissue sample was weighed on ice and homogenized with physiological saline at a ratio of 1:4, *w*/*v*. If the tissue sample was <0.5 g, all tissue was taken; if the tissue sample was 0.5–1.0 g, 0.5 g of tissue sample was taken; if the tissue samples was >1.0 g, 1.0 g of tissue sample was taken. Then, a 100 μL (equivalent to 25 mg) of tissue homogenate was taken and processed in the same manner as shown in “Section 3.5.1”.

### 3.6. Method Validation

The method validation was based on the guidelines set by US Food and Drug Administration (FDA) guidelines [30].

#### 3.6.1. Specificity

The specificity was assessed by comparing the chromatograms of six different lots of rat blank plasma samples, blank plasma (tissue) spiked with anwuligan at LLOQ and IS, and the plasma samples after the administration of anwuligan. There should be no interference at the retention times of the analytes and the IS for blank samples and the retention times should be consistent for spiked samples and actual samples.

#### 3.6.2. Calibration Curve and LLOQ

Calibration curves were constructed using a weighted (1/x^2^) least square linear regression by plotting the peak area ratios of analyte to IS versus the nominal plasma concentrations of anwuligan over the range of 0.5–2000 ng/mL.

The low limit of quantification (LLOQ), defined as the lowest concentration of the calibration curve, should be quantitatively determined with the precision (expressed as RSD, %) within 20% and accuracy (expressed as RE, %) within ± 20%, and the S/N of which should be over 10.

#### 3.6.3. Precision and Accuracy

The intra-day and inter-day precision and accuracy were evaluated by the determination of QC samples at three concentration levels and LLOQ in six replicates on one day and on three consecutive days, respectively. The precision and accuracy were expressed as RSD (%) and RE (%), and expected to be within ± 15% for QC samples.

#### 3.6.4. Extraction Recovery and Matrix Effect

The recovery of the analyte at three QC levels (*n* = 5) was determined by the peak area ratios of extracted analytes to those of the post-extraction samples containing equivalent amounts of targets. The matrix effect was evaluated by comparing the peak areas of the targets in the spike-after-extraction samples with the mean peak areas of it dissolved with the mobile phase at high, medium, and low levels, respectively.

#### 3.6.5. Stability

The stability of anwuligan was evaluated by analyzing five replicates of the samples at three QC levels, including storage in the auto-sampler (4 °C) for 24 h, 4 h exposure at room temperature, three freeze thaw cycles, frozen at −80 °C for 30 days.

#### 3.6.6. Dilution Integrity

At dilution factor 20-fold, the accuracy of the diluted samples for plasma samples (20 ug/mL diluted to 1000 ng/mL) and tissue samples (12.5 ug/mL diluted to 625 ng/mL) was 5.14%–6.21%, and the precision was 5.53%–6.77%, respectively. These results suggested that the study samples can be diluted and maintain adequate accuracy and precision values.

### 3.7. PKs and Tissue Distribution Study Protocols

For the pharmacokinetic study, the rats were fasted for 12 h with free access to water prior to administration. Anwuligan was dissolved in 0.5% DMSO saline (*v*/*v*) to give the intravenous administration solution at the dose of 15 mg/kg and dissolved in 0.5% DMSO saline (*v*/*v*) to give the oral administration solution the dose of 60 mg/kg. For intravenous administration, the approach is using a syringe to deliver the drug into the tail vein; for intragastric administration, the approach is to insert a gastric lavage needle into the mouth of one side of the rat and the drug is slowly injected into the stomach for administration.

A blood sample (0.2 mL) was obtained from the orbital vein and transferred to heparinized tubes at 0.033, 0.083, 0.167, 0.25, 0.50, 0.75, 1, 1.5, 2, 2.5, 3, 4, 8, 10 h after intravenous or oral administration. Six rats are used for each group. The blood samples were immediately centrifuged (12,000× *g*, 10 min) and stored at −80 °C before analysis.

For the tissue distribution study, 16 SD rats received an oral administration at the dose of 60 mg/kg and another 16 SD rats received an intravenous administration at the dose of 15 mg/kg. After that, the tissue samples of heart, liver, spleen, lung, kidney, brain, stomach, and intestine were collected and weighed at 0.5, 1, 2, and 4 h, respectively. For the preparation method of plasma and tissue homogenate samples (see Section 3.5).

### 3.8. Data Analysis

The pharmacokinetic parameters were calculated by a non-compartmental model using the DAS 3.2.8 pharmacokinetic program [31].

## 4. Conclusions

In this study, a rapid, simple, and sensitive UPLC-MS/MS method was developed and validated, following by successfully applied to the pharmacokinetic and tissue distribution investigation of anwuligan after the intravenous or intragastric administration. The method has been validated for the first time in this paper and proved to be able to detect a low concentration of 0.5 ng/mL for anwuligan with liquid-liquid extraction method. The elimination half-life of anwuligan of i.g. administration is relatively shorter than that of i.v. administration. The absolute bioavailability is 16.2%, which represented the existing of the obvious first-pass effect. An enterohepatic circulation was found in the i.g. mode. Anwuligan could be distributed rapidly and widely in different tissues and maintained a high concentration in the liver, suggesting anwuligan might undergo a relative slow elimination and long accumulation in the liver. The developed and validated LC-MS/MS method and the pharmacokinetic study of anwuligan in the present paper would provide data support and reference for the future evaluation of preclinical safety of anwuligan as a candidate drug.

## Figures and Tables

**Table 1:** Calibration curves for anwuligan in biological samples.

Samples	Calibration Curves	Correlation Coefficients (r)	SES *	SEI ^#^	Linear Ranges (ng/mL)	LLOQs (ng/mL)
Plasma	Y = 0.00929 + 0. 000157x	0.996–0.997	6.3 × 10^−4^	0.22	0.5–2000	0.5
Intestine	Y = 0.00378 + 0. 0030x	0.996–0.998	9.6 × 10^−4^	0.37	0.5–2000	0.5
Heart	Y = 0.000419 + 0.0491x	0.995–0.997	8.7 × 10^−4^	0.19	0.5–2000	0.5
Liver	Y = 0.00587 + 0.000116x	0.997–0.999	4.1 × 10^−5^	0.13	0.5–2000	0.5
Spleen	Y = 0.0000987 + 0.0011x	0.994–0.996	7.9 × 10^−4^	0.28	0.5–2000	0.5
Lung	Y = 0.00689 + 0.000892x	0.995–0.997	9.0 × 10^−4^	0.39	0.5–2000	0.5
Kidney	Y = –0.000326 + 0.000131x	0.996–0.998	7.7 × 10^−5^	0.27	0.5–2000	0.5
Stomach	Y = –0.0045 + 0.00088x	0.996–0.997	6.9 × 10^−4^	0.34	0.5–2000	0.5
Brain	Y = –0.0068 + 0.00017x	0.996–0.998	8.2 × 10^−4^	0.26	0.5–2000	0.5

* SES—Standard error of the slope (*n* = 6). ^#^ SEI—Standard error of the intercept (*n* = 6).

**Table 2:** Precision and accuracy of anwuligan in rat plasma and tissues (*n* = 5).

Samples	QC Conc. (ng/mL)	Intra−Day		Inter−Day	
		Precision (RSD, %)	Accuracy (Mean, %)	Precision (RSD, %)	Accuracy (Mean, %)
Plasma	0.5	11.3	−6.7	7.2	5.3
	5	9.6	−5.3	8.3	6.5
	100	5.3	3.5	4.5	−5.2
	1600	8.4	4.9	3.8	5.7
Heart	0.5	7.5	9.5	6.0	10.6
	5	5.4	−7.0	4.0	−9.2
	100	8.5	7.3	7.0	6.7
	1600	8.7	−3.4	4.1	−4.4
Liver	0.5	4.5	−4.7	9.7	−5.5
	5	8.6	6.4	8.8	9.3
	100	7.1	−5.4	3.9	5.9
	1600	5.3	3.2	6.3	−7.4
Spleen	0.5	8.7	−7.9	9.0	−8.9
	5	4.2	4.0	9.3	−4.7
	100	7.1	−4.4	5.7	−7.1
	1600	6.6	6.9	8.8	3.6
Lung	0.5	6.0	7.5	8.0	6.9
	5	4.9	4.4	5.7	−5.0
	100	4.7	−7.1	3.2	3.8
	1600	6.4	−7.2	6.9	6.6
Kidney	0.5	10.9	−11.5	10.6	−12.6
	5	8.4	−4.3	4.9	−5.1
	100	8.5	5.5	9.0	7.6
	1600	5.4	6.7	5.9	−6.8
Brain	0.5	7.0	−9.3	11.3	−8.7
	5	6.3	−5.1	9.7	−5.5
	100	5.5	−4.4	5.3	3.9
	1600	3.9	3.6	4.2	3.4
Intestine	0.5	6.6	8.6	5.9	8.6
	5	4.6	4.3	3.2	7.2
	100	4.0	6.8	8.1	7.6
	1600	8.3	5.4	4.8	3.1
Stomach	0.5	8.7	6.1	6.9	−8.3
	5	7.8	6.6	6.4	−4.0
	100	5.9	−6.2	4.3	3.9
	1600	7.7	−6.2	7.5	−4.4

**Table 3:** Matrix effect and extraction recovery of anwuligan in rat plasma and tissues (*n* = 5).

Samples	QC Conc.(ng/mL)	Matrix Effect		Extract Recovery	
		Mean ± SD (%)	RSD (%)	Mean ± SD (%)	RSD (%)
Plasma	0.5	90.7 ± 2.3	2.6	85.1 ± 2.4	2.9
	5	89.5 ± 7.6	8.6	95.5 ± 5	5.3
	100	96.2 ± 6.3	6.6	109.4 ± 8.5	7.8
	1600	93.1 ± 7.5	8.1	106.4 ± 9.5	8.9
Heart	0.5	90.9 ± 8.8	9.7	86.2 ± 5	5.9
	5	96.6 ± 9.3	9.7	103 ± 2.2	2.1
	100	90.8 ± 4.1	4.6	96.6 ± 9.9	10.3
	1600	84.6 ± 6.3	7.5	84 ± 3.9	4.7
Liver	0.5	93.3 ± 8.3	9.0	109.8 ± 2.4	2.3
	5	89.6 ± 7.2	8.1	106.3 ± 6.4	6.1
	100	85.1 ± 6.0	7.1	114 ± 2.8	2.5
	1600	84.5 ± 9.2	11.0	96.9 ± 9.6	9.9
Spleen	0.5	89.2 ± 6.5	7.3	104.6 ± 4.9	4.7
	5	91.9 ± 4.2	4.6	108.8 ± 3.9	3.6
	100	86.3 ± 7.3	8.5	111.3 ± 2.3	2.1
	1600	96.8 ± 6.5	6.7	90.7 ± 8.8	9.7
Lung	0.5	87.5 ± 5.8	6.7	94.1 ± 3.9	4.2
	5	93.8 ± 2.2	2.4	93.3 ± 8.1	8.8
	100	95.0 ± 9.0	9.5	101.9 ± 9.9	9.7
	1600	88.3 ± 9.8	11.1	102.2 ± 2.8	2.7
Kidney	0.5	86.7 ± 3.9	4.6	95.9 ± 2.4	2.6
	5	87.2 ± 8.7	10.1	114.5 ± 8.4	7.4
	100	87.4 ± 9.8	11.2	105.2 ± 5	4.8
	1600	90.5 ± 4.3	4.8	84.5 ± 4.5	5.4
Brain	0.5	88.5 ± 3.7	4.2	102.5 ± 5.8	5.7
	5	95.8 ± 4.5	4.7	84.8 ± 4.9	5.8
	100	95.1 ± 7.0	7.4	103.6 ± 5.4	5.3
	1600	85.5 ± 5.7	6.7	106.9 ± 6.4	6.0
Intestine	0.5	85.8 ± 3.9	4.6	93 ± 4.7	5.1
	5	86.0 ± 2.8	3.4	104.9 ± 5.4	5.2
	100	84.6 ± 8.7	10.3	102 ± 9.8	9.6
	1600	92.8 ± 8.1	8.8	92.8 ± 7.1	7.6
Stomach	0.5	91.8 ± 6.9	7.5	114.5 ± 8.1	7.1
	5	88.0 ± 2.4	2.8	96.7 ± 2.8	2.9
	100	87.8 ± 9.0	10.3	93.8 ± 9	9.6
	1600	91.7 ± 3.7	4.0	102 ± 3.4	3.4

**Table 4:** Stability results for anwuligan in rat plasma and tissues homogenates of rats (*n* = 5).

Samples	QC Conc.(ng/mL)	Short-Term (at Room Temperature for 4 h)	Autosampler 4 °C for 24 h	Three Freeze-Thraw Cycles	Storage at −80 °C for 30 d
Plasma	5	110.4 ± 3.2	114.4 ± 10.7	110.0 ± 4.7	106.7 ± 3.3
	1600	94.2 ± 10.4	85.2 ± 5.9	86.0 ± 4.3	99.0 ± 5.6
Heart	5	95.0 ± 10.4	90.3 ± 10.6	107.1 ± 4.5	85.2 ± 4.5
	1600	94.8 ± 5.2	102.6 ± 7.8	111.8 ± 7.3	96.0 ± 3.3
Liver	5	97.3 ± 4.8	91.6 ± 8.0	105.6 ± 9.1	103.2 ± 7.7
	1600	87.1 ± 8.9	87.0 ± 7.5	106.7 ± 9.5	86.4 ± 6.3
Spleen	5	98.5 ± 10.2	100.9 ± 5.3	98.6 ± 10.5	110.0 ± 8.2
	1600	105.6 ± 5.3	87.6 ± 5.1	102.6 ± 7.7	114.9 ± 6.2
Lung	5	112.4 ± 3.7	97.2 ± 3.5	88.6 ± 7.6	111.1 ± 4.8
	1600	87.2 ± 9.7	93.6 ± 4.0	112.9 ± 9.7	92.8 ± 7.5
Kidney	5	95.7 ± 9.9	95.2 ± 6.3	88.7 ± 6.4	86.4 ± 8.4
	1600	99.8 ± 9.1	97.1 ± 6.2	104.3 ± 9.3	112.2 ± 6.3
Brain	5	93.2 ± 7.4	104.5 ± 4.2	90.0 ± 5.0	112.8 ± 7.7
	1600	104.1 ± 4.0	106.2 ± 3.3	106.9 ± 8.7	112.5 ± 7.1
Intestine	5	103.8 ± 4.9	95.5 ± 6.6	87.1 ± 4.1	95.1 ± 9.6
	1600	111.6 ± 10.1	110.3 ± 7.1	108.3 ± 7.4	113.9 ± 4.9
Stomach	5	90.2 ± 8.0	99.9 ± 3.2	88.8 ± 4.3	105.9 ± 7.5
	1600	104.0 ± 10.1	109.4 ± 9.8	107.2 ± 9.4	89.3 ± 8.7

**Table 5:** Non-compartmental pharmacokinetic parameters of anwuligan.

Pharmacokinetic Parameters	Intravenous	Intragastric
C_max_ (ng/mL)	8310 ± 910	810 ± 190
t_1/2_ (h)	3.20 ± 1.09	5.17 ± 1.82
AUC_0–t_ (ng/mL∙h)	16,700 ± 7700	10,700 ± 2800
AUC_0–∞_ (ng/mL∙h)	16,800 ± 8500	11,400 ± 3600
MRT_0–∞_ (h)	3.71 ± 0.96	10.02 ± 2.04
CL (L/h/kg)	0.89 ± 0.32	5.26 ± 1.39
V_d_ (L/kg)	4.1 ± 1.1	39.3 ± 12.6
F (bioavailability, %)	-	16.2

## Supplementary Materials

The following are available online, Table S1: Pharmacology of anwuligan.
