# Supplementary material for: Pharmacokinetics and Tissue Distribution of Anwuligan in Rats after Intravenous and Intragastric Administration by Liquid Chromatography-Mass Spectrometry
Source: Molecules. 2019 Dec 20;25(1):39. doi: 10.3390/molecules25010039 (PMC6983174; doi:10.3390/molecules25010039)
Supplement: Supplementary file 1 [file molecules-25-00039-s001.pdf]

Table S1 Pharmacology of anwuligan.

Pharmacological effects	Details	Cell lines/model	Dosage of administration	Application	Ref
Gastro-protective and Anti-cariogenic function	Anti-bacterial property<br>*Streptococcus mutans*<br>*Lactobacillus*<br>*Actinomyces*	vegetative cells and spores of Bacillus cereus	MIC=4 μg/mL	in vitro	1
anti-cancer drug	Cancer chemopreventive effect<br>Anti-carcinogenic activity	HL-60 cell	100μM	in vitro	2
Hepato-protection	Human hepatoma cell protection<br>Protective effect against hepatotoxicity<br>MAPK signaling pathway	HepG2 cell	0.5 to 5μM	in vitro	3
Dermalogical protection	Melanogenesis inhibition<br>Photoaging and inflammation attenuation	B16F10 melanoma cells	1 to 10 μM	in vitro	4
Anti-oxidant and anti-inflammatory	Inhibition of lipid peroxidation (LPO)<br>Free radical scavenging<br>Reduction of proinflammatory cytokine<br>Inhibit degranulation of histamine	RBL-2 H3 cells	5, 10, or 20 μ M	in vitro	5
anti-diabetes	Insulin secretagogue action<br>Lipid metabolism and insulin sensitivity<br>Upregulation of Adipocyte gene expression	COS-7 cells, 3T3-L1 preadipocytes, SK-HEP1 hepatocytes, HepG2<br>hepatocytes, and C2C12 skeletal myoblast cells<br>Obese diabetic mice (db/db) and C57BL/6J	EC_{50}= 4.221<br>0.01 to 25 mol/l<br>10 mg . kg^{-1} day^{-1}~25	in vivo.<br>in vitro	6

		mice	mg . kg^{-1} day^{-1} .		
Neuro-protective activities	Learning and memory enhancement<br>Glutamate-induced neurotoxicity attenuation<br>Attenuation of ROS production<br>Microglia activation reduction<br>MAPK signaling<br>NF-jB activity<br>ER-stress resistance and insulin-resistance<br>Elevation of acetylcholine levels	Fisher-344 rat	10 mg/kg	in vivo	7

Reference

1. Rukayadi, Y., K. Lee, S. Han, S. Kim, and J.K. Hwang. 2009.Antibacterial and sporicidal activity of Macelignan isolated from nutmeg (Myristica fragrans Houtt.) against Bacillus cereus. Food Science and Biotechnology 18: 1301–1304.

2. Park, B.Y., B.S. Min, O.K. Kwon, S.R. Oh, K.S. Ahn, T.J. Kim, D.Y.Kim, K. Bae, and H.K. Lee. 2004. Increase of caspase-3 activity by lignans from Machilus thunbergii in HL-60 cells. Biological &/and Pharmaceutical Bulletin 27: 1305–1307.

3. Sohn, J.H., K.L. Han, J.H. Choo, and J.K. Hwang. 2007. Macelignan protects HepG2 cells against tert-butylhydroperoxide-induced oxidative damage. BioFactors 29: 1–10.

4. Choi, E.J., Y.G. Kang, J. Kim, and J.K. Hwang. 2011. Macelignan inhibits melanosome transfer mediated by protease-activated receptor-2 in keratinocytes. Biological &/and Pharmaceutical Bulletin 34: 748–754.

5. Han, Y.S., M.S. Kim, and J.K. Hwang. 2012. Macelignan inhibits histamine release and inflammatory mediator production in activated rat basophilic leukemia mast cells. Inflammation 35. doi:10.1007/s10753-012-9490-1.

6. Han, K.L., J.S. Choi, J.Y. Lee, J. Song, M.K. Joe, M.H. Jung, and J.K. Hwang. 2008. Therapeutic potential of peroxisome proliferators-activated receptor-alpha/gamma dual agonist with alleviation of endoplasmic reticulum stress for the treatment of diabetes. Diabetes 57: 737–745.

7. Cui, C.A., D.Q. Jin, Y.K. Hwang, I.S. Lee, J.K. Hwang, I. Ha, and J.S. Han. 2008. Macelignan attenuates LPS-induced inflamma-tion and reduces LPS-induced spatial learning impairments in rats. Neuroscience Letters 448: 110–114.
